# The Corset

**Published:** 1891-01

**Authors:** R. A. Chudleigh


### THE CORSET.

Respiration is maintained by two sets of muscles ; one set called the intercostals lie between every pair of ribs ; in the other set are comprised the diaphragm and the abdominal muscles which assist it. There is an old and wide-spread belief that while males breathe chiefly with the diaphragm, women for obvious physiological reasons breathe almost entirely with the ribs; leaving the diaphragm nearly quiescent; insomuch that to the detective eye a heaving chest is supposed to reveal a woman under all disguises. This fact seems to be established by a series of "stethograms," or respiration curves, taken over various areas of the chest, which were recently exhibited at Birmingham. The cause, however, of the difference between male and female respiratory movements was shown to be, not as has been supposed, a natural and beneficent variation in the respiratory mechanism, but simply and solely the use of corsets. The movements of respiration are freely communicated to the whole solid mass of the liver; and the effect of this natural and perpetual liver massage must be to maintain the onward flow of bile—a matter of the utmost importance to health—while, on the other hand, the biliary arrangements must become dislocated even to stagnation, when a person so compresses her liver with a corset as to arrest the respiratory pumping. In Oriental women, and in all women who habitually discard corsets, these respiratory movements are not less free, and, in some cases, are actually more free, than in most men. Hence we may conclude that there is no natural difference in the respiratory mechanism of the sexes; and whatever difference does exist is due to dress and not to nature.—R. A. CHUDLEIGH, *Wimborne.*

